# Association Between Celiac Disease and Uncontrolled Hemoglobin A1c Levels in Type 1 Diabetes Pediatric Patients

**DOI:** 10.1155/pedi/5225066

**Published:** 2026-02-11

**Authors:** Amy Lin, Michelle Jankowski, Shirley Qu, Virginia Uhley, Michael Brennan, Ramin Homayouni

**Affiliations:** ^1^ Oakland University William Beaumont School of Medicine, Rochester, Michigan, USA, oakland.edu; ^2^ Corewell Health Research Institute, Royal Oak, Michigan, USA; ^3^ Corewell Health William Beaumont University Hospital, Royal Oak, Michigan, USA

**Keywords:** celiac disease, glycemic control, hemoglobin A1c, pediatric endocrinology, type 1 diabetes

## Abstract

**Background:**

Celiac disease (CD) occurs in ~6% of individuals with type 1 diabetes (T1D) and may complicate glycemic control due to conflicting dietary needs. Prior studies show mixed results regarding the impact of CD on Hemoglobin A1c (HbA1c), especially in pediatric populations. This study evaluates whether CD is associated with suboptimal glycemic control in pediatric patients with T1D.

**Methods:**

This retrospective chart review analyzed pediatric patients (<18 years) diagnosed with T1D between 2012 and 2023 across Corewell Health East. Patients were identified via ICD‐10 codes and stratified by CD status and glycemic control (controlled HbA1c <7% vs. uncontrolled HbA1c ≥7%). Statistical analyses include chi‐square or Fisher’s exact tests for categorical variables, Wilcoxon tests for continuous variables, and logistic regression for multivariable analysis.

**Results:**

Among 2,203 pediatric patients with T1D, 101 (4.6%) had CD. Patients with both conditions were younger at T1D diagnosis (median age 9 vs. 12 years, *p* < 0.0001) and had more HbA1c measurements. A higher proportion of CD patients had uncontrolled diabetes (89.1% vs. 73.8%, *p* = 0.0006). CD was independently associated with uncontrolled HbA1c (adjusted OR: 2.59; 95% CI: 1.37–4.90; *p* = 0.003) after adjusting for age, sex, and race. Younger age and Black race were also associated with higher odds of uncontrolled diabetes.

**Conclusion:**

CD is significantly associated with poorer glycemic control in pediatric patients with T1D, independent of age, race, and sex. These findings suggest the need for closer monitoring, individualized dietary counseling, and targeted interventions in this high‐risk group.

**Summary**

• Children with both type 1 diabetes and celiac disease are more likely to have poor glycemic control, highlighting the added burden of managing dual autoimmune conditions.

## 1. Introduction

The co‐occurrence of type 1 diabetes (T1D) and celiac disease (CD) is well‐documented, with studies estimating that ~6% of individuals with T1D also develop CD [1]. Prevalence estimates vary widely depending on population and diagnostic criteria, with serology‐based studies in children reporting rates between 1.4% and 24.5% [2]. The overlap between T1D and CD is largely attributable to shared autoimmune susceptibility, particularly within the human leukocyte antigen (HLA) class II region. The association is strongest among carriers of HLA‐DQ2 and HLA‐DQ8 haplotypes, which confer increased risk for both T1D and CD [3]. The current American Diabetes Association (ADA) guidelines recommend routine screening for CD in individuals with T1D to ensure early detection and appropriate management [4].

One of the key challenges in managing patients with both conditions is dietary adaptation, particularly regarding glycemic control. While a gluten‐free diet (GFD) is the only effective treatment for CD, gluten‐free products often have a high glycemic index (GI), which contradicts the recommended low‐GI diet for T1D management. This dietary conflict necessitates specialized nutritional guidance to balance carbohydrate intake while maintaining adequate blood glucose levels [5]. A recent review demonstrated that implementing a GFD in children with both T1D and CD does not appear to worsen glycemic control. Although some earlier studies reported a transient increase in HbA1c during the first year following CD diagnosis, more recent analyses and meta‐analyses have found no significant long‐term differences in HbA1c, insulin requirements, or hypoglycemic events between children with and without CD [6].

Adherence to a GFD is often poor among patients with both T1D and CD, with studies indicating that non‐adherence can contribute to suboptimal glycemic control [7, 8] and therefore an increased risk of microvascular and macrovascular complications [9]. Additionally, children with both T1D and CD face greater barriers to adhering to a GFD compared to those with CD alone [10, 11]. From a health‐economics perspective, early screening and integrated management of T1D and CD can be cost‐effective, as they may reduce acute complications, lower long‐term healthcare expenditures, and improve overall quality of life [12].

Existing studies offer mixed findings on the relationship between CD and glycemic control. Some have reported that adults with both conditions have higher Hemoglobin A1c (HbA1c) levels compared to those with T1D alone [13], while others have found no significant differences [14–16]. Conversely, one study reported lower HbA1c levels in patients with coexisting CD and T1D [17].

In light of these variable results, our study aims to evaluate the association between CD and HbA1c levels in pediatric patients with T1D, providing new insights into how CD diagnosis influences glycemic control in this high‐risk population.

## 2. Methods

### 2.1. Study Design and Population

This was a retrospective cross‐sectional study of pediatric patients (<18 years) diagnosed with type 1 diabetes (T1D; ICD‐10 code E10.x) who received care within the Corewell Health East system in metropolitan Detroit, Michigan, between January 1, 2012, and December 31, 2023. Of 24,479 patients with a T1D diagnosis during the study period, 2575 were aged <18 years. After excluding those without an available HbA1c measurement (*n* = 372), 2203 pediatric patients were included in the final analysis. Patients were categorized according to the presence or absence of CD and by glycemic control status based on mean HbA1c values. The study inclusion criteria were (1) age <18 years at any visit during the study period, (2) diagnosis of T1D recorded in the medical record, and (3) at least one HbA1c measurement available. Patients were excluded if they had missing demographic data or incomplete diagnostic information.

### 2.2. Data Collection and Variables

All data were extracted from the institution’s electronic medical record (EMR) data warehouse (Epic Clarity). Demographic variables included age, sex, race, and ethnicity. Clinical variables included age at T1D diagnosis, number of HbA1c measurements, mean HbA1c level, and CD status. CD was identified by using ICD‐10 codes K90.x found in encounter billing, medical history, and problem lists. Serologic/biopsy confirmation was not consistently available in the EMR. Because of inconsistencies in the timing of HbA1c measurements relative to the CD diagnosis, all available HbA1c values for each patient were extracted, and the mean HbA1c per patient was used as the primary analytic variable. Socioeconomic indicators (e.g., family income, insurance type, or socioeconomic status) were not consistently available and therefore were not included in the analysis. In addition, because documentation of GFD adherence was inconsistently available, it was not included in the quantitative analysis.

### 2.3. Outcomes

The primary outcome for this study was glycemic control, defined according to the ADA standards of care [18]. Uncontrolled diabetes was defined as a mean HbA1c ≥7% while controlled diabetes was defined as HbA1c <7%. Secondary outcomes included the number of HbA1c measurements per patient and the association between demographic factors and uncontrolled diabetes.

### 2.4. Statistical Analysis

Descriptive statistics were reported as medians with interquartile ranges (IQR) for continuous variables and frequencies with percentages for categorical variables. Between‐group comparisons were performed using the chi‐square or Fisher’s exact test for the categorical variables and the Wilcoxon rank‐sum test for continuous variables. Multivariable logistic regression was used to estimate adjusted odds ratios and 95% confidence intervals (CI) for factors associated with uncontrolled diabetes. The model included CD status, age at T1D diagnosis, sex, and race as covariates. All statistical tests were two‐tailed, and *p*  < 0.05 was considered statistically significant. Analysis was performed using SAS 9.4 (SAS Institute Inc., Cary, NC, USA).

### 2.5. Ethical Considerations

This study was approved by the Corewell Health Institutional Review Board and determined to be exempt under Category 4, as it involves secondary research using identifiable health information in compliance with HIPAA and institutional regulations. All data were de‐identified before analysis.

## 3. Results

### 3.1. Characteristics of T1D Patients

A total of 2203 pediatric patients with T1D were included in the analysis (Table 1). The majority of T1D patients were male (54.2%), White race (70.3%), non‐Hispanic/Latino ethnicity (77.2%), and had a median age of 12 (IQR: 8–15). On average, T1D patients had eight (IQR 2–17) HbA1c measurements during the study period with a mean HbA1c of 8.36 (±2.29 SD).

**Table 1:** Characteristics of pediatric patients with type 1 diabetes treated at Corewell Health East between 2012 and 2023, stratified by the presence of celiac disease and glycemic control status (defined as HbA1c <7% vs. ≥7%).

Variable	All	Celiac disease			Controlled HbA1c (<7%)		
		No	Yes	*p*‐Value ^∗^	Yes	No	*p*‐Value ^∗^
Sample, *n* (row %)	2203	2102 (95.4)	101 (4.6)	—	561 (25.5)	1642 (74.5)	—
Age, median (IQR)	12 (8–15)	12 (8–15)	9 (5–12)	<0.0001	13 (10–15)	11 (8–14)	<0.0001
Female, *n* (col %)	1009 (45.8)	956 (45.5)	53 (52.5)	0.1681	234 (41.7)	775 (47.2)	0.0243
Race, *n* (col %)	—	—	—	0.0277	—	—	<0.0001
White/Caucasian	1524 (70.3)	1442 (68.6)	82 (81.2)	—	412 (73.5)	1112 (67.7)	—
Black/AA	373 (17.2)	364 (17.3)	9 (8.9)	—	52 (9.3)	321 (19.5)	—
Other	271 (12.5)	262 (12.5)	9 (8.9)	—	87 (15.5)	184 (11.2)	—
ND	35	34	1	—	10	25	—
Ethnicity (col %)	—	—	—	0.1471	—	—	<0.0001
Arab or middle eastern descent	171 (8.1)	162 (7.7)	9 (8.9)	—	72 (13.9)	99 (6.7)	—
Hispanic/Latino	81 (3.8)	81 (3.9)	0 (0.0)	—	32 (6.2)	49 (3.3)	—
Non‐Hispanic/Latino	1632 (77.2)	1549 (73.7)	83 (82.2)	—	349 (67.3)	1283 (83.6)	—
Other	229 (10.8)	221 (10.5)	8 (7.9)	—	80 (15.4)	149 (9.7)	—
ND	90	89	1	—	28	62	—
Average of all A1c values, mean (SD)	8.36 (2.29)	8.36 (2.32)	8.49 (1.51)	0.5563	5.54 (0.69)	9.33 (1.79)	<0.0001
Number of A1c values, median (IQR)	8 (2–17)	8 (2–16)	14 (7–27)	<0.0001	2 (1–4)	11 (5–20)	<0.0001
HbA1c ≥ 7% (col %)	1642 (74.5)	1552 (73.8)	90 (89.1)	0.0006	561 (0)	1642 (100)	—
Celiac disease (col %)	101 (4.6)	0 (0)	101 (100)	—	11 (2.0)	90 (5.5)	0.0006

^∗^ *p*‐Values were determined using chi‐square or Fisher’s exact test for categorical variables and Wilcoxin test for continuous variables.

### 3.2. Characteristics of T1D Patients With CD

Among the entire study cohort, 4.6% (*n* = 101) had a concurrent diagnosis of CD. Patients with both T1D and CD were significantly younger at the time of T1D diagnosis compared to those without CD (median age: 9 vs. 12 years; *p*  < 0.0001). On average, patients with both conditions had a significantly (*p*  < 0.0001) higher number of HbA1c measurements (14 vs. 8). There were no significant differences in sex and ethnicity distribution. CD was more prevalent among White patients (5.4%) with T1D, and less so among Black/African American patients (2.4%) and other racial groups (3.3%; *p* = 0.0277). Other racial groups include patients who self‐identified as “Other” or as Asian, American Indian or Alaska Native, or Native Hawaiian or Pacific Islander. The mean HbA1c did not differ significantly between T1D patients with or without CD (8.49% vs. 8.36%; *p* = 0.5563), but a significantly (*p* = 0.0006) higher proportion of T1D patients with CD had uncontrolled diabetes (HbA1*c*≥7% (53 mmol/mol): 89.1% vs. 73.8%).

### 3.3. Characteristics of T1D Patients With Uncontrolled HbA1c

Among the entire study cohort, 74.5% (*n* = 1642) had uncontrolled diabetes. These patients were significantly younger at the time of T1D diagnosis (median age: 11 vs. 13 years; *p*  < 0.0001) and had a greater median number of HbA1c values (11 vs. 2; *p*  < 0.0001) compared to those with controlled diabetes. Additionally, there were significant differences in racial (*p*  < 0.0001) and ethnic (*p*  < 0.001) groups when comparing controlled and uncontrolled T1D patients. Lastly, a significantly higher proportion of uncontrolled T1D were female (*p* = 0.0243).

### 3.4. Factors Associated With Uncontrolled T1D

Since the univariate analysis showed that a higher proportion of T1D patients with CD had uncontrolled diabetes, we conducted a multivariable logistic regression to examine whether CD was an independent predictor of glycemic control. After adjusting for age, race, and sex, the presence of CD remained significantly associated with uncontrolled diabetes (adjusted OR: 2.59; 95% CI: 1.37–4.90; *p* = 0.003), as shown in Figure 1.

Figure 1Risk factors associated with uncontrolled type 1 diabetes (HbA1c ≥7%). Forest plot of logistic regression results depicting (A) unadjusted and (B) adjusted odds ratios (OR) and 95% confidence intervals for factors significantly (*p*  < 0.05) associated with uncontrolled type 1 diabetes (T1D).

**Figure:**
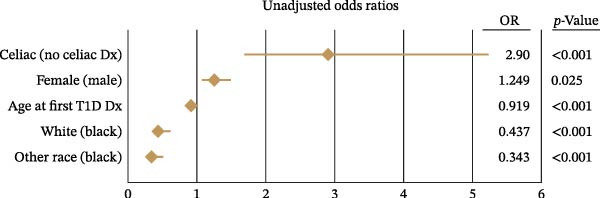
(A)

**Figure:**
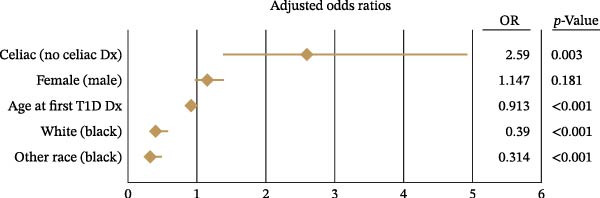
(B)

Additionally, we found that each year of age when T1D diagnosis occurred decreased the odds of uncontrolled diabetes by 8.7% (adjusted OR: 0.913; 95% CI: 0.891–0.936; *p* < 0.001). Compared to Black patients, White (adjusted OR: 0.390; 95% CI: 0.281–0.541) and other racial groups (adjusted OR: 0.314; 95% CI: 0.210–0.469) were significantly less likely to have uncontrolled diabetes (*p* < 0.001 for both). Although females had slightly higher odds of uncontrolled diabetes in the unadjusted model, this difference was not statistically significant after adjustment (adjusted OR: 1.15; *p* = 0.181).

## 4. Discussion

In this retrospective study, we found that 4.6% of pediatric patients with T1D had a concurrent diagnosis of CD, which aligns with previously reported prevalence rates in this population [1, 19]. Our study builds on the existing literature in several important ways. First, while prior work has evaluated glycemic outcomes in mixed pediatric or adult cohorts, our analysis focuses specifically on a large pediatric population drawn from a diverse metropolitan health system, providing updated real‐world data in a group for whom evidence remains limited. Second, unlike earlier studies that have often relied on single HbA1c measurements, we evaluated mean HbA1c values over a prolonged period, offering a more stable assessment of glycemic control. Third, we observed that children with both T1D and CD were diagnosed with diabetes at a significantly younger age, a finding consistent with previous reports [19, 20]. Importantly, we demonstrate a statistically significant and independent association between CD and uncontrolled diabetes, even after adjusting for key demographic variables. Taken together, our findings contribute to the ongoing debate regarding the impact of CD on glycemic control in patients with T1D. While prior studies have reported mixed results, often concluding that CD does not significantly affect HbA1c [6], our data provide real‐world evidence that the presence of CD is independently associated with a higher likelihood of suboptimal glycemic control in pediatric patients. Importantly, this association persists even after adjustment for key demographic factors, suggesting that CD may influence glycemic outcomes in routine clinical practice more than previously appreciated.

### 4.1. CD and Glycemic Control

Patients with both T1D and CD had a significantly higher number of HbA1c measurements (14 vs. 8, *p*  < 0.0001), possibly reflecting more frequent monitoring due to the complexity of dual disease management. Notably, although the mean HbA1c did not differ significantly between groups, a significantly higher proportion of patients with both T1D and CD had suboptimal glycemic control (HbA1c ≥7%) compared to those without CD (89.1% vs. 73.8%, *p*  < 0.0006). Using multivariable logistic regression, we found that T1D patients with CD had more than twice the odds of having uncontrolled HbA1c (adjusted OR: 2.59, 95% CI: 1.37–4.90), after adjusting for potential confounders such as age, race, and sex. These findings suggest that while average glycemic control may appear similar between groups, children with coexisting CD are disproportionately represented among those struggling to meet recommended HbA1c targets. This pattern may be obscured in studies relying solely on mean HbA1c values and highlights the importance of examining clinically meaningful thresholds of glycemic control.

Previous work by Bashiri et al. [21] reported a higher prevalence of uncontrolled diabetes in adult patients with CD compared to those without, though the difference did not reach statistical significance (*p* = 0.09). Our study helps clarify this relationship by demonstrating a statistically significant and independent association between CD and uncontrolled diabetes. Other studies, which included both pediatric and adult patients, have found no significant difference in HbA1c levels between those with coexisting CD and T1D and those with T1D alone [14, 16]. However, these studies primarily included patients with long‐standing, treated CD and may therefore reflect outcomes after the initiation of a GFD. In contrast, our pediatric cohort likely includes patients with varying durations of CD and with varying adherence to treatment. Individuals with newly diagnosed or untreated CD are particularly vulnerable to glycemic instability due to developmental challenges, inconsistent adherence, and the added burden of dietary restrictions [7]. In this context, inconsistent adherence to a strict GFD, particularly in children and adolescents, may contribute to greater glycemic variability and a higher risk of suboptimal control. This distinction may help explain why our findings differ from prior reports and highlights the importance of considering treatment status and adherence when interpreting the relationship between CD and glycemic outcomes.

Interestingly, a recent study by James et al. [17] using registry data from Australia, it was reported that patients ages 16–25 years with coexisting T1D and CD had slightly lower HbA1c levels than those with T1D alone. This finding, though seemingly contradictory, may reflect differences in disease duration, timing of CD diagnosis, or access to multidisciplinary care and dietary support, further highlighting the heterogeneity of this population.

Several mechanisms may explain the association between CD and suboptimal glycemic control. CD can impair nutrient absorption, complicate insulin dosing, and impose additional dietary restrictions, which may disrupt carbohydrate counting and mealtime consistency. Chronic intestinal inflammation may also contribute to a degree of insulin resistance, further challenging glycemic management and adherence. Additionally, the burden of managing two chronic autoimmune conditions may affect both patients and caregivers, influencing overall disease management [10].

Our study reinforces the need for routine CD screening in patients with T1D. Beyond glycemic control, CD has been linked to increased risks of diabetes‐related microvascular and macrovascular complications [22–24]. Early treatment with GFD has been associated with improved glycemic outcomes in T1D patients [25–28]. Our results suggest that without effective dietary management, CD may remain a clinically relevant contributor to poor glycemic control, emphasizing the need for early diagnosis, dietary education, and ongoing adherence support.

While the coexistence of T1D and CD is well‐documented, large‐scale data examining how CD impacts glycemic control in pediatric populations remains limited [29]. By providing updated real‐world data from a large metropolitan health system, our study adds to the growing body of evidence suggesting that CD may meaningfully influence glycemic outcomes in children with T1D, particularly outside the context of strictly controlled dietary adherence. Further studies are needed to evaluate the long‐term impact of a GFD on glycemic control, growth, and quality of life in children with coexisting T1D and CD.

### 4.2. Effect of Age, Sex, and Race

After adjusting for key demographic factors, age at T1D diagnosis and race were significantly associated with uncontrolled diabetes. Each additional year of age was associated with an 8.7% decrease in the odds of uncontrolled diabetes, indicating that younger patients are at higher risk for poor long‐term glycemic outcomes. This is consistent with a prior study showing that younger age at T1D diagnosis predicts worse glycemic control [30]. Regarding race, Black patients had significantly higher odds of uncontrolled diabetes compared to White and other race categories, even after adjustment of age, sex, and CD status. These findings align with previous reports demonstrating persistent racial disparities in HbA1c and diabetes outcomes, which are thought to be driven by differences in access to care, diabetes technology use, social determinants of health, and systemic inequities [31, 32].

Ninety‐seven percent of pediatric patients in Michigan are covered by health insurance [33], so the racial disparities in diabetes control are unlikely to stem from lack of access to care alone. Instead, factors such as food insecurity, transportation barriers, and limited health literacy likely contribute to poorer disease management and persistently worse outcomes among African American youth. To address these disparities, future efforts should include screening for and mitigating food insecurity, improving transportation access, and enhancing culturally tailored diabetes education to improve health literacy. Although female sex was associated with slightly higher odds of uncontrolled diabetes in unadjusted models, this difference was not statistically significant after adjustment, suggesting it may be explained by age and racial differences. Nonetheless, some studies have reported that young females with T1D may experience higher HbA1c levels at diagnosis and over time [34], warranting further investigation.

### 4.3. Limitations

This retrospective study is based on data from the EMRs, which are subject to potential misclassification, incomplete documentation, and selection bias. For example, information regarding serological confirmation of CD as well as adherence to a GFD was not available consistently in the EMR. As the analysis was limited to a single health system, generalizability may be limited. Patients with CD had more frequent HbA1c measurements, introducing possible detection bias. While we adjusted for age, race, and sex in our analysis, other important confounding factors, such as socioeconomic status, GFD adherence, insulin regimens, and frequency of endocrinology follow‐up, were not captured and may influence glycemic control.

## 5. Conclusion

In this retrospective study of pediatric patients with T1D, concurrent diagnosis of CD was significantly associated with higher odds of uncontrolled diabetes. Our findings add to the limited pediatric literature by demonstrating that CD may contribute to greater glycemic instability and increased clinical monitoring needs in this population. These findings suggest possible challenges in managing pediatric patients with T1D and CD. Additionally, younger age at T1D diagnosis and Black race were significant predictors of poor glycemic control, addressing disparities that warrant targeted attention. Taken together, these results highlight the importance of heightened clinical awareness, routine screening for CD in children with T1D, and the development of tailored management strategies to support children and families navigating both conditions. Healthcare providers must deliver more tailored, effective care for patients with both CD and T1D to improve their long‐term health outcomes.

## Author Contributions

Amy Lin, Ramin Homayouni, Michael Brennan and Virginia Uhley conceptualized the study and designed the methodology. Michelle Jankowski and Shirley Qu collected and curated the data and performed the statistical analysis. Amy Lin and Ramin Homayouni drafted the manuscript. Michael Brennan and Virginia Uhley reviewed and edited the manuscript.

## Funding

The authors received no specific funding for this work.

## Disclosure

Portions of this work were presented at the American Diabetes Association 85th Scientific Sessions, June 20−23, 2025. All authors approved the final version of the manuscript.

## Conflicts of Interest

Dr. Michael Brennan is a paid consultant for Novo Nordisk, Boehringer Ingelheim, and Bayer. Dr. Homayouni is a paid consultant of SimplePractice and is a co‐founder and equity holder of Quire Inc. All other authors declare no conflicts of interest.

## Data Availability

The data that support the findings of this study are available on request from the corresponding author. The data are not publicly available due to privacy or ethical restrictions.
